# Elevated Omentin Serum Levels Predict Long-Term Survival in Critically Ill Patients

**DOI:** 10.1155/2016/3149243

**Published:** 2016-10-27

**Authors:** Mark Luedde, Fabian Benz, Jennifer Niedeggen, Mihael Vucur, Hans-Joerg Hippe, Martina E. Spehlmann, Florian Schueller, Sven Loosen, Norbert Frey, Christian Trautwein, Alexander Koch, Tom Luedde, Frank Tacke, Christoph Roderburg

**Affiliations:** ^1^Department of Internal Medicine III, University Clinic of Schleswig-Holstein, Schittenhelmstrasse 12, 24105 Kiel, Germany; ^2^Department of Medicine III, University Hospital RWTH Aachen, Pauwelsstrasse 30, 52074 Aachen, Germany

## Abstract

*Introduction.* Omentin, a recently described adipokine, was shown to be involved in the pathophysiology of inflammatory and infectious diseases. However, its role in critical illness and sepsis is currently unknown.* Materials and Methods.* Omentin serum concentrations were measured in 117 ICU-patients (84 with septic and 33 with nonseptic disease etiology) admitted to the medical ICU. Results were compared with 50 healthy controls.* Results.* Omentin serum levels of critically ill patients at admission to the ICU or after 72 hours of ICU treatment were similar compared to healthy controls. Moreover, circulating omentin levels were independent of sepsis and etiology of critical illness. Notably, serum concentrations of omentin could not be linked to concentrations of inflammatory cytokines or routinely used sepsis markers. While serum levels of omentin were not predictive for short term survival during ICU treatment, low omentin concentrations were an independent predictor of patients' overall survival. Omentin levels strongly correlated with that of other adipokines (e.g., leptin receptor or adiponectin), which have also been identified as prognostic markers in critical illness.* Conclusions.* Although circulating omentin levels did not differ between ICU-patients and controls, elevated omentin levels were predictive for an impaired patients' long term survival.

## 1. Introduction

The visceral adipose tissue (VAT) was identified as critical source of bioactive proteins—so-called adipokines—during inflammatory diseases [1, 2]. By directly regulating hyperglycemia, glucose intolerance, and insulin resistance, adipokines function as prognostic indicators in several diseases [3, 4]. However, in ICU patients, only few data on the regulation, kinetics, and correlation of omentin serum levels with the course of the disease including end points (e.g., death) are available.

Besides well-established adipokines such as leptin or resistin, omentin was recently identified as a new member of the adipokine family [5]. Omentin is encoded by 2 different genes, omentin-1 and omentin-2, and is mainly expressed and secreted by the visceral adipose tissue [5].* In vitro*, treatment of human adipocytes with omentin was associated with an enhanced activation of the Akt pathway, leading to an increased glucose uptake upon stimulation with insulin. Moreover, pretreatment with omentin negatively modulated the TNF-*α* dependent phosphorylation and activation of inflammatory signaling pathways such as p38 or JNK, leading to an impaired production of cytotoxic stress molecules [6–8].* In vivo*, elevated omentin serum concentrations were found in patients with obesity, diabetes, inflammatory bowel diseases, rheumatoid arthritis, asthma, coronary artery disease, and heart failure. Several studies on patients with coronary artery disease even revealed a prognostic function of omentin levels [9–12].

In this study we examined concentrations of circulating omentin in a large cohort of patients from a medical ICU. These data might not only establish omentin as a biomarker in critical illness but also help to further define the role of omentin in systemic inflammation and sepsis.

## 2. Materials and Methods

### 2.1. Study Design and Patient Characteristics

117 patients that were consecutively admitted between February 2012 and August 2014 to our medical ICU were included in the study. The cohort consisted of 70 males and 47 females, respectively. Patient characteristics are shown in Table 1. The study protocol was approved by the local ethics committee (Ethics Committee of the University Hospital Aachen, RWTH-University, Aachen, Germany) and conducted in accordance with the ethical standards laid down in the Declaration of Helsinki. Written informed consent was obtained from the patient, his or her spouse, or the appointed legal guardian. Patient information and samples were acquired prospectively, and follow-up was performed as recently described [13]. Presence of septic disease was defined according to the criteria defined in the third consensus definition of sepsis [14]. All other patients were categorized as nonsepsis patients [15, 16]. Omentin serum levels in critically ill patients were compared with 50 healthy blood donors (35 male, 15 female, median age 37 years, and range between 18 and 67)* free* of chronic diseases like diabetes and cardiac and pulmonary diseases and with normal values for blood counts, C-reactive protein, and liver enzymes.

### 2.2. Measurements of Omentin Serum Levels by ELISA

Blood collection was performed at the day of admission to the ICU and after 3 days of treatment. Sample handling and analysis of routine laboratory and experimental parameters were performed as described previously [15, 17, 18]. We determined omentin serum levels by using a commercially available enzyme immunoassay (ELISA, Ray Biotech, Norcross, GA, USA) according to manufacturers' instructions.

### 2.3. Statistical Analysis

Statistics applied in this analysis have recently been described [15, 17, 18]. In summary, data are expressed as median and range. The Mann–Whitney *U* test and for multiple comparisons the Kruskal-Wallis-ANOVA were used. Correlation analysis was performed by using the Spearman correlation test, and the prognostic value of the variables was tested by univariate and multivariate analysis in the Cox regression model. Kaplan-Meier curves were plotted to display the impact on survival. Finally, ROC curves were generated by plotting sensitivity against 1 − specificity. All statistical analyses were performed with SPSS version 23 (SPSS, Chicago, IL, USA) [19, 20].

## 3. Results

### 3.1. Omentin Serum Concentrations in Critically Ill and Sepsis Patients

**Figure 1:**
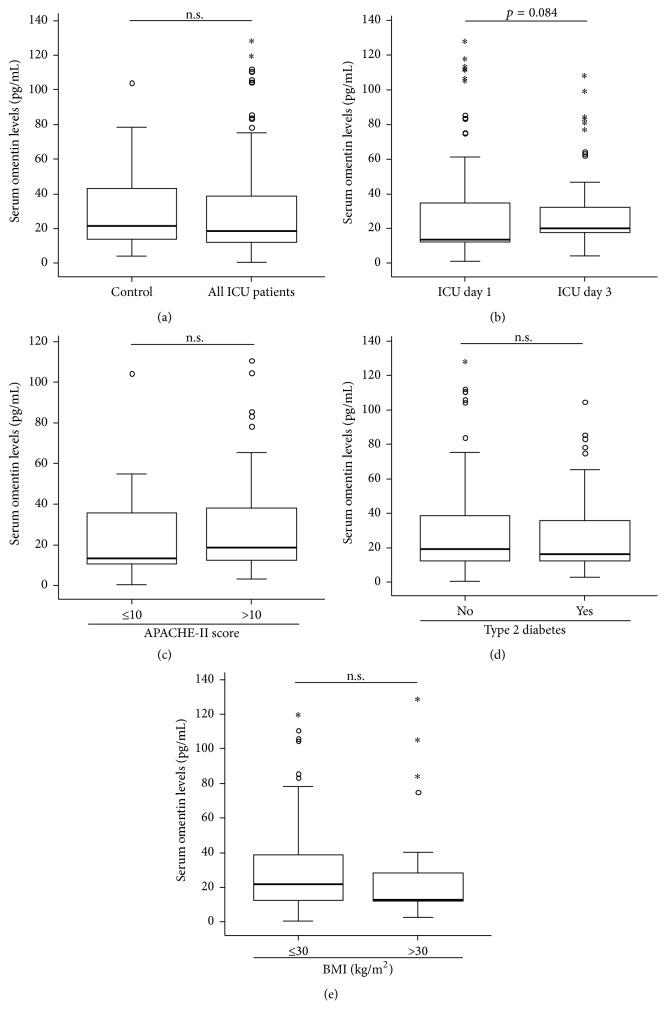
Serum omentin concentrations of critically ill patients at ICU admission and after 3 days of ICU treatment. (a) Omentin levels were determined by ELISA in patients at admission to the ICU (*n* = 117) and in healthy controls (*n* = 50). (b) Omentin levels were measured after three days of ICU treatment (*n* = 79) and compared to levels at admission to the ICU as well as to omentin concentrations of healthy controls. (c) Omentin levels at admission to the ICU were independent of the severity of critical illness* (APACHE-2 score at admission to the ICU)*. (d) Omentin levels were measured in patients with or without type 2 diabetes. (e) Omentin levels were measured in patients with or without obesity. Box plots are displayed, where the bold line indicates the median per group, the box represents 50% of the values, and horizontal lines show minimum and maximum values of the calculated nonoutlier values; asterisks and open circles indicate outlier values.

Based on recent data suggesting a potential value of omentin serum levels in inflammatory diseases, we aimed to determine the role of circulating omentin in critically ill patients. Therefore, omentin levels were measured in a large and well defined cohort of 117 ICU patients (Table 1) and in 50 healthy volunteers. Notably, omentin serum levels were similar between ICU patients and healthy controls (Figure 1(a)). For 79 out of 117 patients, longitudinal measurements were available. Similar to omentin measurements at admission to the ICU, omentin concentrations at day 3 did not differ to healthy controls or to omentin concentrations at day 1 (Figure 1(b)). Accordingly, omentin concentrations were not related to the disease severity (APACHE-2 score at admission to the ICU; Figure 1(c)). Considering the link between circulating omentin and metabolic diseases [21], we further analyzed whether obese patients or patients with type 2 diabetes displayed alterations in their omentin levels. However, at least in our cohort of critically ill patients, levels of circulating omentin were independent of these characteristics (Figures 1(d) and 1(e)). Notably, omentin concentrations were also independent of patient's age or gender (Table 1).

**Figure 2:**
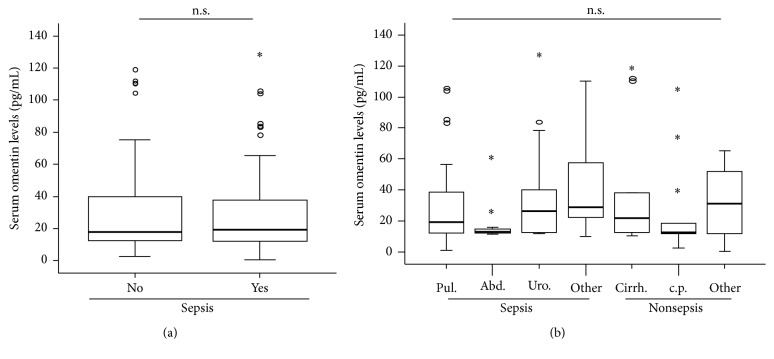
Serum omentin concentrations in patients with sepsis. (a) ICU patients with sepsis (*n* = 84) displayed similar omentin levels compared to patients without sepsis (*n* = 33, *U* test). (b) Levels of circulating omentin were not different in patients with different etiologies of critical illness. Box plots are displayed, where the bold line indicates the median per group, the box represents 50% of the values, and horizontal lines show minimum and maximum values of the calculated nonoutlier values; asterisks and open circles indicate outlier values.

Our cohort of 117 critically ill patients comprises 84 patients with sepsis and 33 patients with nonseptic etiology of disease. No differences in omentin levels between patients with sepsis and those that did not fulfill sepsis criteria became apparent (Figure 2(a)). Moreover, omentin concentrations did not correlate with markers of inflammation or infection such as C-reactive protein (CRP), procalcitonin (PCT), and interleukin-6 (IL-6) (Table 2). We further performed a subgroup analysis to understand whether a deregulation of omentin might be specific for certain disease etiologies. Among the 84 patients with septic disease, 53 were categorized as pulmonary sepsis, 11 as abdominal sepsis, 4 as urogenital sepsis, and 16 as septic diseases with a different/unknown focus. Among the nonseptic patients, 13 suffered from cardiopulmonary diseases, 7 suffered from decompensated liver cirrhosis, and 13 had another etiology of critical illness. By comparing serum omentin concentrations between these different groups we did not observe any differences (Figure 2(b)), thus demonstrating that omentin does not represent a general marker for critical illness and sepsis.

### 3.2. Elevated Omentin Concentrations Predict an Increased Mortality Rate

**Figure 3:**
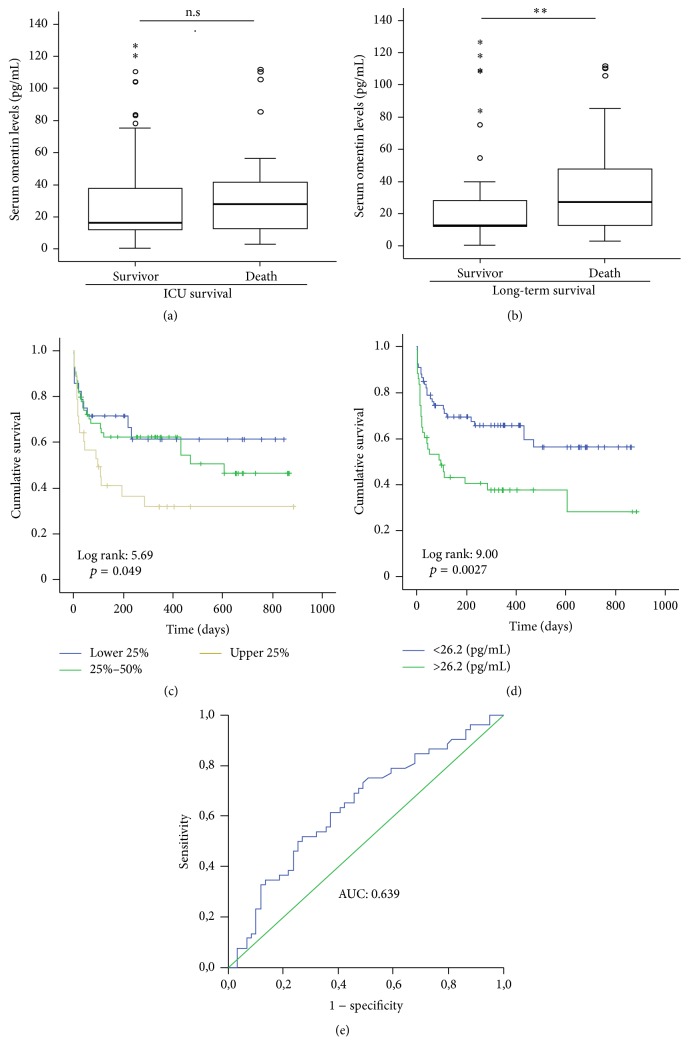
Prediction of long-term mortality by concentrations of circulating omentin. (a) Serum omentin concentrations were measured in patients that died during ICU treatment and survivors. (b) Omentin concentrations analyzed in patients that survived in the long-term follow-up and patients who did not survive (*U* test). (c + d) Kaplan-Meier curve analysis with respect to patients' serum omentin concentrations. (e) ROC curve analysis on the value of Omentin measurements for prediction of patients prognosis. Box plots are displayed, where the bold line indicates the median per group, the box represents 50% of the values, and horizontal lines show minimum and maximum values of the calculated nonoutlier values; asterisks and open circles indicate outlier values.

Several reports suggested an association between elevated omentin concentrations and the probability of survival in patients with inflammatory diseases. To identify an association between circulating omentin and the prognosis of critically ill patients, we compared the omentin levels in survivors and patients that did not survive. In accordance with our previous results, there was no difference between the patients that survived and the patients that died (Figure 3(a)).

Within our cohort of critically ill patients, 25% died on the ICU and an additional 20% died during long-term follow-up, median follow-up of 353 days (range 29−800 days). We therefore tested whether omentin levels at admission to ICU were predictive for the patients' long-term prognosis. Strikingly, patients that survived during the long-term follow-up period had significantly lower omentin concentrations than those patients that succumbed to death (*p* = 0.009; Figure 3(b)). We next performed Kaplan-Meier curve analysis and Cox regression analysis to determine the impact of elevated omentin levels on patient survival in our cohort of critically ill patients. Interestingly, patients with elevated omentin levels (4th quartile) displayed a significantly higher mortality compared to the other patients (Figure 3(c)). We determined the optimal threshold with the highest Youden Index for omentin levels predicting patient long-term survival. The best sensitivity and specificity scores for survival were obtained when an omentin cutoff level of 26.2 ng/mL was used. Based on this value, we performed Kaplan-Meier survival analysis, showing significantly impaired survival in critically ill patients with omentin levels >26.2 pg/mL compared to patients with lower omentin concentrations (Figure 3(d)). Of note, the mortality within the “omentin high” group was 63% compared to 36% within the “omentin low” group (*p* = 0.007; Pearson Chi-Square test). Finally, multivariate Cox regression analyses identified omentin as an independent prognostic parameter in critically ill patients.

**Figure 4:**
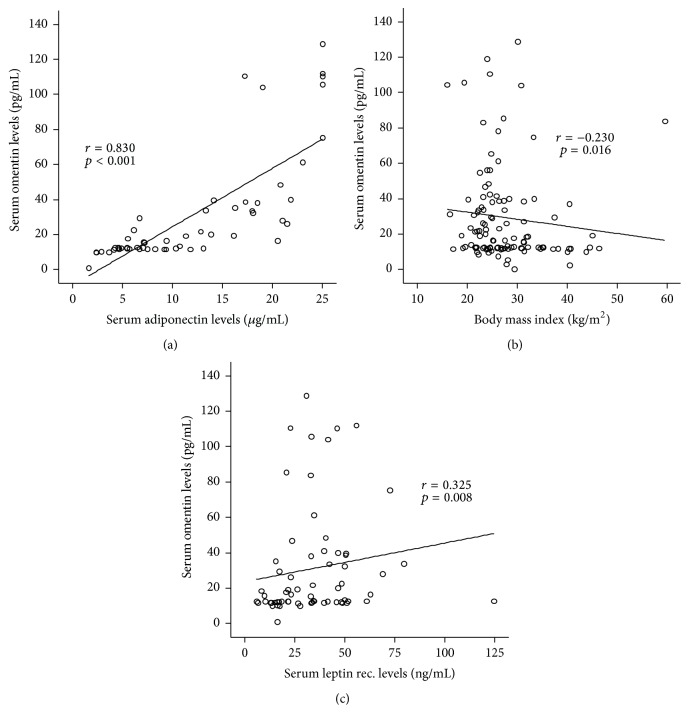
Omentin serum levels are correlated with metabolic parameters of critically ill patients. Serum adiponectin concentrations, body mass index (BMI), and leptin receptor concentrations in critically ill patients are correlated with omentin levels.

In an attempt to identify factors regulating omentin serum levels in patients with critical illness, we next applied correlation analyses between omentin serum concentrations and a broad set of clinical routine as well as experimental parameters. As mentioned, there was no association between omentin levels and markers for inflammation and infection. Furthermore, in conformity with our data on the lack of an association between omentin and ICU survival, we did not uncover a link between omentin concentrations and markers for organ failure or an unfavorable prognosis on the ICU. Moreover, omentin levels at admission did not correlate with insulin demand or concentrations of C-peptide both at admission to the ICU and after three days of treatment. However, omentin at admission was correlated with adiponectin (*r* = 0.830, *p* < 0.001), leptin receptor (*r* = 0.325, *p* = 0.008), and also the patient's body mass index ((*r* = 0.230, *p* = 0.016); Figure 4), indicating that metabolically active adipose tissue is the primary source of omentin in critically ill patients.

## 4. Discussion

The traditional concept that scales down adipose tissue to being a storage pool for excess energy has been challenged by multiple studies defining a novel role of adipose tissue as a complex hormonally active system that regulates metabolism and inflammation [22]. Cytokines secreted by adipose tissue are called adipokines. Dysregulation of adipokines has been linked to many disease states such as diabetes and obesity [11] and also to cardiovascular diseases [23], bone diseases [24], carcinogenesis [25], chronic kidney disease [26], sepsis [27], and even bipolar disorder [28]. Remarkably, in our cohorts of critically ill patients due to sepsis and different entities like cardiogenic shock and liver failure, adipokines like adiponectin [29], resistin [18], and leptin [4] have been shown to associate with patient survival, underlining a critical role of adipokines in the progression of sepsis and nonseptic critical illness.

Our study determines serum concentrations of omentin in a well characterized cohort of critically ill patients upon admission to the medical ICU. Omentin is a recently identified Ca^2+^ dependent adipokine that has been denominated as a “good” adipokine, promoting beneficial metabolic effects [30]. Initially described in 2005 [31], it was also referred to as intelectin-1 or “endothelial lectin” [32] due to its close resemblance to lectin in function and structure. It is encoded by two genes, omentin-1 and omentin-2, with omentin-1 being the major circulating form. Thus we here refer to omentin-1 as omentin. This molecule consists of 313 amino acids and is mainly expressed in adipose tissue [30]. With high affinity for galactofuranosyl residues [31], it has been proposed to be in the recognition of specific bacterial components [33].

Omentin expression has been demonstrated to be associated with obesity. While circulating levels of omentin are reduced by obesity [7], weight reduction leads to an increase of omentin concentrations [34]. Likewise, omentin seems to play a protective role in cardiovascular disease [35]. These protective effects of omentin in both obesity and cardiovascular disease seem to be based on disease-specific effects, for example, an insulin sensitizing effect in obesity [36] and modulation of NO synthase function in the coronary endothelium [37]. On the other hand, omentin seems to exert its effects by interfering with a condition that combines diseases like obesity, diabetes, coronary artery disease, heart failure, pulmonary disease, and chronic kidney disease: a state of chronic low-grade inflammation [30]. Mechanistically, omentin inhibits inflammation via several signaling pathways, for example, by inhibiting tumor necrosis factor-alpha (TNF-*α*) and NF*κ*B induced expression of intracellular adhesion molecule-1 (ICAM-1) and vascular cell adhesion molecule 1 (VCAM-1) [37]. We analyzed critically ill patients to assess if this comprehensive anti-inflammatory role of omentin might also have a functional role in this collective.

Of note, we did not detect alterations of omentin serum levels in critically ill patients compared to healthy controls. Previous work on serum leptin levels in critically ill patients found an equal effect. Similar to omentin-1, the leptin and also leptin receptor concentrations did not differ between patients with critical illness and healthy controls [4]. Consistent with serum leptin levels, subgroup analyses revealed no significant difference in sepsis and nonsepsis patients. Remarkably, both molecules proved to be an unfavorable indicator of overall cumulative long-term survival, and higher serum levels of omentin were correlated to impaired survival. In contrast to these similarities, both molecules also show important discrepancies in critically ill patients. While leptin serum levels significantly correlated with the incidence of obesity and/or diabetes in these patients, omentin levels did not show a significant correlation with these parameters. In contrast to omentin, serum leptin levels inversely correlated with serum PCT [4]. These opposing results underline the complex regulation and functional differentiation of adipokines. As a possible explanation for these disparate effects, one might speculate that leptin might rather act as a “mediator” of obesity or diabetes related prognostic implications, while omentin-1 exerts effects independent of obesity and diabetes. In this regard, it may be worth mentioning that a negative prognostic impact of obesity has not been conclusively demonstrated in critically ill patients [38, 39].

As stated above, a close association of omentin-1 and inflammatory pathways has been proposed [30]. Surprisingly, omentin serum levels did not correlate with markers of acute inflammation or infection like CRP or procalcitonin (PCT). The correlation of these markers with acute critical illness and short-term prognosis has been conclusively demonstrated before [40, 41]. In this regard, it may seem coherent that omentin serum levels were not differentially regulated in critically ill patients versus healthy controls at ICU admission and did not correlate with short-term prognosis. The fact that high omentin levels correlated with a declined long-term prognosis may shed light on an issue that has not been sufficiently addressed so far: the impact of persistent low-grade inflammation on long-term prognosis of ICU patients [42]. Inflammation and especially acute systemic inflammatory reaction syndrome (SIRS) have been extensively studied in the acute phase of critical illness, but it is unclear how many patients have evidence of ongoing inflammation in the recovery phase [42]. It is known that the majority of ICU patients exhibit elevated CRP levels at the time of hospital discharge [43]. However, the value of elevated CRP levels as a predictor of ICU outcome is at least unclear [44]. Compared to C-reactive protein, omentin might prove a more promising target in the poorly apprehended setting of post-ICU course of disease, linking chronic inflammation and long-term outcome. Of course, further molecular studies on the complex interactions of omentin and other adipokines during inflammation and critical disease are needed to elucidate their functional role in this context. In this regard, it is still unclear if omentin exerts beneficial effects in critical illness. The highly significant association of elevated omentin serum levels and poor prognosis of ICU patients may reflect either direct malign effects of omentin in critical illness or—more likely—a compensatory upregulation of a generally beneficial [30] molecule. Of note, in line with chronic inflammation, the impact of diabetes [45] and obesity on the outcome of ICU patients is not fully understood. In fact, improved survival of ICU patients with mild to moderate obesity has been proposed, referred to as the obesity paradox in the ICU [39]. Linking obesity and altered metabolism to inflammation, omentin might be a central regulator of the complex network of molecular pathway modulating the course of disease during and after ICU treatment.

### 4.1. Limitations of This Study

Our report is not a case control study. Thus, patients and controls are not fully matched regarding sex and age. Moreover, since the panel of clinical information for the control group was not as complete as it was for the patients group, we cannot fully exclude further imbalances between both groups. However, in our analysis, serum omentin levels were not correlated to patients' sex or age; thus it seems unlikely that at least these factors have biased our analysis. Finally, the multiple groups used in analysis of ICU patient subgroups consequently lead to relatively small numbers in each group. Thus, larger studies combining prospective clinical trials and experimental animal models are needed to finally define the role of omentin measurements in a collective of patients with a still unacceptably poor prognosis.

## Figures and Tables

**Table 1:** Study population.

Parameter	All patients
Number	117
Sex (male/female)	70/47
Age median (range) [years]	66 (18–90)
APACHE-II score median (range)	18 (3–40)
SAPS2 score median (range)	43 (9–80)
ICU days median (range)	9 (1–137)
Death during ICU *n* (%)	24.8%
Ventilation time median (range) [h]	121 (0.0–2966.0)
Preexisting diabetes *n* (%)	32.8%
HbA1c [%]	5.8 (4–12.60)
BMI [kg/m^2^]	26.09 (15.9–59.5)
WBC median (range) [×10^3^/*μ*L]	12.7 (0.1–208)
CRP median (range) [mg/dL]	116 (<5–230)
Procalcitonin median (range) [*μ*g/L]	1.0 (0.0–125.2)
Interleukin-6 median (range) [pg/mL]	73 (0–6100)
*Albumin median (range) [g/dL]*	*2.8 (0.8–4.4)*
*INR median (range)*	*1.16 (0.94–4.62)*
*AST median (range) [U/L]*	*41 (10.0–2591)*
*GLDH median (range) [U/L]*	*4.15 (0.5–720)*
*Creatinine median (range) [mg/dL]*	*1.4 (0.1–10.7)*
*Urea median (range) [mg/dL]*	*73 (5.0–455)*
*Adiponectin median (range) [μg/mL]*	*10.28 (15.98–25.00)*
*Leptin receptor median (range) [ng/mL]*	*33.02 (5.74–124.46)*
*Ghrelin median (range) [pmol/L]*	*17.4 (5–113.8)*

APACHE, Acute Physiology and Chronic Health Evaluation; CRP, C-reactive protein; ICU, intensive care unit; SAPS, simplified acute physiology score; WBC, white blood cell count.

**Table 2:** Correlations of omentin serum concentrations at admission day with other laboratory markers at admission to the ICU.

Parameter	*r*	*p*
*Markers of liver function*		
Albumin	−0.127	0.276
INR	0.136	0.147
AST	−0.014	0.890
Bilirubin	0.085	0.367
GLDH	−0.103	0.317

*Markers of inflammation*		
CRP	−0.087	0.356
Procalcitonin	−0.074	0.517

*Markers of renal function*		
Creatinine	0.061	0.515
Urea	0.124	0.183

*Metabolic markers*		
Adiponectin	0.830	<0.001
Leptin receptor	0.325	0.008
BMI	−0.230	0.016
Ghrelin	0.220	0.080
HbA1c	−0.265	0.043
Insulin demand d1	−0.068	0.613
Insulin demand d3	−0.016	0.874
C-peptide d1	0.229	0.081
C-peptide d3	0.109	0.502

*r*, correlation coefficient; *p*, *p* value; *r* and *p* values by Spearman rank correlation.
